# In vitro anti-biofilm properties of the peel of fruite wall of acorn against Streptococcus mutans

**DOI:** 10.3205/dgkh000449

**Published:** 2023-09-27

**Authors:** Zahra Chavak, Nahid Mahdian, Iraj Pakzad, Mohammad Reza Soltani, Behzad Badakhsh, Sobhan Ghafourian

**Affiliations:** 1 Department of Microbiology, Faculty of Medicine, Ilam University of Medical Sciences, Ilam, Iran; 2 Department of Operative Dentistry, School of Dentistry, Ilam University of Medical of Sciences, Ilam, Iran; 3 Department of Gastroenterology, Faculty of Medicine, IlamUniversity of Medical Sciences, Ilam, Iran

**Keywords:** fruite wall of acorn, ethanolic extract, antibiofilm efficacy, S. mutans, cytotoxicity

## Abstract

Dental caries is a multi-factorial infectious disease. The primary cause is dental plaque, a complex of biofilm. It was postulated that the ethanolic extract of fruite wall of acorn may represent a new substance to prevent caries. Hence, the study was performed to evaluate the effect of ethanolic extract of fruite wall of acorn against biofilm formation by *Streptococcus* *mutans*, which is associated with dental plaque. The cytotoxicity of the ethanolic extract was determined against Vero cells resulting in an inhibitory concentration of 50 (IC_50_) of 55 µg/ml. After bacterial collection, different concentrations under the IC_50_ from the extract were evaluated against biofilm formation of *S. mutans*. 3 µg/ml of the extract inhibited the biofilm formation of *S. mutans*, and 1 to 3 µg/ml caused a decrease in *gtfB* and *brpA* biofilm-production genes. This study showed the potency of the ethanolic extract of fruite wall of acorn against biofilm formation by *S. mutans*.

## Abstract (de)

Zahnkaries ist eine multifaktorielle Infektionskrankheit. Die Hauptursache sind dentale Plaques, ein komplexer Biofilm. Auf der Basis der Hypothese, dass der ethanolische Extrakt der Schale der Eichel eine neue Substanz sein könnte, Zahnkaries zu verhindern, wurde die Wirkung des ethanolischen Extrakts gegen die Biofilmbildung von *Streptococcus mutans*, der mit Karies assoziiert ist, geprüft. Für die Zytotoxizität des ethanolischen Extrakts gegen Vero-Zellen wurde eine IC_50_ von 55 µg/ml ermittelt. Nach Entnahme der Bakterien wurde die Wirkung verschiedener Konzentrationen des Pflanzenextraktextrakts unterhalb der IC_50_ auf die Biofilmbildung von *S. mutans* geprüft. 3 µg/ml des Extrakts hemmten die Biofilmbildung von *S. mutans* und 1 bis 3 µg/ml induzierten eine Abnahme der bildungbildenden Gene *gtfB* und *brpA*. Die Untersuchung bestätigte die Hypothese der Wirksamkeit des ethanolischen Pflanzenextrakts der Eichel gegen die Biofilmbildung von *S. mutans*.

## Introduction

Human dental caries, a microbial disease, is one of the most prevalent oral infectious diseases in developing countries [1]. A major etiological agent of dental caries, *Streptococcus (S.) mutans*, resides primarily in biofilms that form the dental plaques on the tooth surfaces [2]. Other etiological factors consist of acid production and sophisticated environmental stress adaptation that contribute to the initiation and progression of dental caries [3]. Studies of *S. mutans* have focused on understanding the molecular mechanisms of forming robust biofilms on tooth surfaces, rapidly metabolizing a wide variety of carbohydrates obtained from the host diet, and surviving numerous (and frequent) environmental challenges encountered in oral biofilms. Thus, *S. mutans* served as a model organism for dental biofilms [2]. The biofilm regulatory protein A *(brpA* gene) plays a major role in biofilm formation. A deficiency of *brpA* adversely affects the fitness and diminishes the virulence of* S. mutans* [4]. *brpA* was found to encode a novel protein of 406 amino acid residues. A strain carrying an insertionally inactivated copy of *brpA* formed longer chains than did the parental strain, aggregated in liquid culture, and was unable to form biofilms as shown by an in vitro biofilm assay [5]. The Beta glucosyltransferase *(gtfB)* gene catalyzes the formation of soluble and insoluble glucans. Insoluble glucans cause an increase the pathogenicity of oral biofilm by promoting the initial adherence of *S. mutans* on tooth surfaces. Therefore, inhibition of the *brpA* and *gtfB* genes is one of the strategies to control biofilm formation and the plaque-related diseases [6], [7], [8], [9].

The gold standard solution for inhibiting dental plaque is the antiseptic agent chlorhexidine digluconate (CHG) [10]. However, due to its widespread use, an increase in type I and type IV allergies against CHG has been observed [11]. After previous topical application of CHG, 0.4% and 1% of patients with contact allergy reacted positively in the patch test [12], [13]. The prevalence of perioperative allergic reactions was 9–10% in the United Kingdom, Denmark, and Belgium [14], [15], [16]. Rarely, anaphylactic reactions with the risk of anaphylactic shock are also caused. A recent survey reported 252 cases of perioperative anaphylaxis triggered by CHG [17]. Especially critical is the development of tolerance and resistance to CHG with the development of new antibiotic resistances. These are explained, for instance, by the presence of the antiseptic resistance genes *qacA, qacB* and *qacC*, the staphylococcal multidrug resistance (smr) gene and multi-resistance plasmids [18]. Because of the side effects of CHG and the risk of developing cross-resistance to antibiotics, the use of CHG should be carefully considered and avoided for indications for which other antiseptics with comparable efficacy and no risk of causing resistance can be considered.

In search of alternatives for oral cavity antiseptics, some research has focused on finding medicinal-plant extracts as safe agents for biofilm inhibition [19]. Adisc-diffusion assay of propanolic extracts of Camelina oil and seeds found them to be antimicrobially effective [20]. The antimicrobial efficacy of cold-pressed oregano (Origanum vulgare) oil suggests that it could be used economically as a valuable natural product with novel functional properties in food, cosmetics and pharmaceutical industries [21]. Another interesting herb is Quercus infectoria galls with long history in traditional Chinese and Uyghur medicine for treatment of diarrhea, hemorrhage, skin disease, and many other human ailments [22]. The fruite wall of acorn is widely used throughout western Iran, especially in the Ilam province, to treat inflammation and diarrhea. Ethanol extracts of fruite wall of acorn are antimicrobially effective against *Klebsiella pneumoniae, Escherichia coli, Staphylococcus aureus, Salmonella typhi* and *Pseudomonas aeroginosa* (minimal inhibitory concentration 10, 10, 5, 15 resp. 15 µg/ml). Experimental infection in rats by *K. pneumoniae, E. coli, S. typhi* and *P. aeruginosa* was completely inhibited, while positive control rats died after five days [23]. Although the fruite wall of acorn is common against various diseases, the information about inhibition of biofilm formation is not sufficient. Based on the foregoing, it was postulated that fruite wall of acorn may represent a new substance to prevent dental caries. Hence, the present study was performed to evaluate the efficacy of ethanolic extract of fruite wall of acorn against biofilm formation of *Streptococcus mutans*.

## Material and methods

### Bacterial strain and culture conditions

Sixty dental caries samples were collected from healthy volunteers at Ilam University of Medical Sciences (with patients’ consent). Samples were grown on mitis-salivarius (MS) agar and Chocolate agar (Condalab). The plates were incubated for 48 hours at 37°C under 5% CO_2_. Biochemical tests were performed to identify *S. mutans*, including Gram staining, catalase, oxidase, lactose, trehalose, fermenting of mannose and mannitol. A molecular assay was performed to amplify the *gtfB* and *brpA* genes.

### DNA extraction and PCR

DNA was extracted using a boiling method. The extracted DNA was stored at –20°C until used. To amplify the partial sequence of the *gtfB* and *brpA* genes, specific primers were used (Table 1 (Tab. 1)). The amplification was performed by PCR.

**Figure 1:**
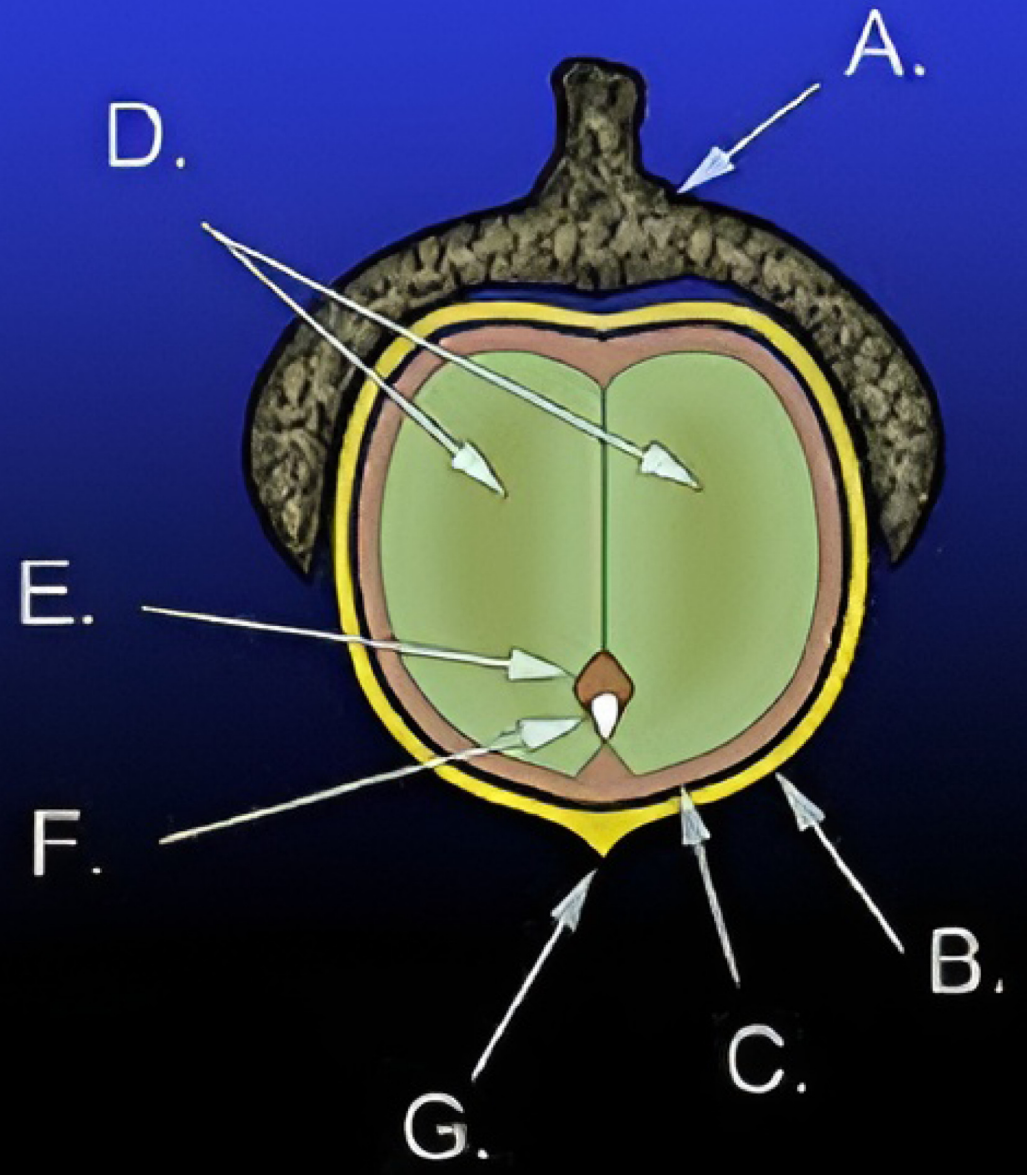
Diagram of the anatomy of an acorn: A.) Cupule B.) Pericarp (fruit wall) C.) Seed coat (testa) D.) Cotyledons (2) E.) Plumule F.) Radicle G.) Remains of style. Together D., E., and F. make up the embryo [27].

Plant collection and preparation of herbal extract: fruite wall of acorn were collected from the Ilam forests in the west of Iran. The fruite wall of acorn were isolated and air-dried. Ethanol extract of dried fruite wall of acorn was also prepared (Figure 1 (Fig. 1)).

### Cell culture

African green monkey kidney (Vero) cell lines were obtained from the Pasture Institute of Iran. The cells were grown in FBS and 1% penicillin-streptomycin at 37°C under microaerophilic conditions. Cells were subcultured after they formed a monolayer on the flask. The cells were detached by treating with trypsin-EDTA (0.25% trypsin containing 0.01% EDTA) for 10 min and then by adding a complete medium to inhibit the reaction. 

### Toxicity assay

After cell counting, 5,000 to 6,000 cells were poured into each well of the microplate. After 48 hours, the cells adhered to the plate, after which they were exposed to different concentrations of the extract of the fruite wall of acorn. After 48 hours, the wells were stained bt trypan blue and the IC_50_ was determined. 

### Qualitative evaluation of biofilm

Bacteria were grown overnight in Mueller-Hinton broth supplemented with 5% of glucose. The culture was diluted at 1:200 and incubated overnight in stationary U-bottom 96-well microliter plates at 37°C. After washing twice with phosphate-buffered saline, the plates were dried in an inverted position at room temprature and stained with 0.1% crystal violet.

### Anti-biofilm activity

The effect of the fruite wall of acorn extract on biofilm formation of all isolates was evaluated by using the microtiter plate method. Two-fold serial dilutions of the extract were made in sterile 96-well microplates containing 190 µl Mueller-Hinton broth with and without 5% sucrose per well. 10 µl of fresh bacterial suspension (0.5 McFarland) was added to each well. The tested concentration range was from 1 µg/ml to 40 µg/ml. Two growth controls [(bacteria+broth+extract) and (bacteria+broth+extract 5% sucrose)], a medium-control (only broth) and a blank control (broth+extract) were included. After incubation at 37°C for 48 hours, the biofilm biomass was assayed using the crystal violet staining assay. The absorbance was measured at 570 nm.

### RNA extraction and cDNA synthesis

Total RNA was extracted using a Hybrid-R kit (Gene All, South Korea) according to the manufacturer’s instructions. The quantity of the extracted RNA was analyzed using a NanoDrop spectrometer. Synthesis of cDNA was performed using a cDNA Synthesis Kit (GeneAll, South Korea), according to the manufacturer’s instructions.

### Real-time PCR assay

Real-time PCR was used to quantify *gtfB* and *brpA*. All primers for real-time PCR were designed with Genescript (Table 2 (Tab. 2)). Real-time PCR amplification was performed on the CFX96 Real-Time (Bio Rad, USA). Amplification was performed in a 25 µl reaction mixture containing 12.5 µl SYBR green PCR Master Mix (Applied Biosystems), 2 µl template cDNA, 2 µl forward and reverse primers (10 mM each) and 8.5 µl deionized water. 16S rRNA *S. mutans* was applied as refrence gene [24].

## Results

### S. mutans determination by phenotypic and molecular assay confirmation

**Figure 2:**
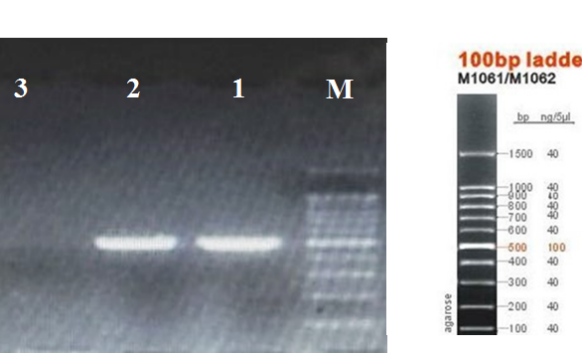
PCR for *gtfB* gene (M=marker 100 bp, 1 and 2=*gtfB* 519 bp, 3=negative control)

**Figure 3:**
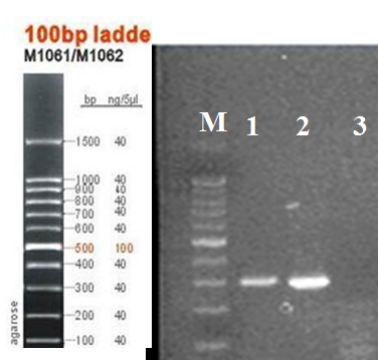
PCR for *brpA* gene (M=marker 100 bp, 1 and 2=*brpA* 299 bp, 3=negative control)

Of the 60 samples collected, 30 samples of bacteria were catalase and oxidase negative, but mannose fermenter, lactose fermentation tests and salicin were positive. Final confirmation was performed by evaluation of the *gtfB* gene. The presence of *brpA *was also confirmed by PCR (Figure 2 (Fig. 2) and Figure 3 (Fig. 3)).

### Biofilm formation

Biofilm formation assay in the normal environment was done with sucrose 5% and no sucrose in Muller-Hinton culture medium. The results showed an increase of biofilm formation in the presence of sugar (Table 3 (Tab. 3)).

### Toxicity and antibiofilm properties of the herbal extract

The IC_50_ was determined with 55 µg/ml. Therefore, different concentrations of the extract below IC_50_ were evaluated against biofilm formation of *S. mutans*, Showing that 3 µg/ml could inhibit the biofilm formation of *S. mutans*.

### Real-time PCR assay

The expression of the genes in different concentrations of the extract was evaluated. The mean gene expression at dilutions of 1 to 3 µg/ml of the herbal extract showed a decrease of expression of *gtfB* and *brpA* (Table 4 (Tab. 4), Table 5 (Tab. 5)).

The level of gene expression before exposure to the extract is considered as one-fold. Then, after exposure to the extract at different concentrations, the level of expression was compared with the “one-fold”.

As shown in Table 4 (Tab. 4) and Table 5 (Tab. 5), the expression rate is different in samples with different concentations of the herbal extract. The result can be attributed to the antibiofilm activity of the extract. 

.

## Discussion

*Streptococcus mutans* is the most important and in dental plaque, it has been proven to cause caries. *S. mutans* causes the loss of tooth enamel by producing lactic acid and use of sucrose. According to the literature, tooth decay (dental caries) is present in many of people in the society, the attachment of bacteria is the first and most important factor in the process of tooth decay. Dental caries is promoted by not regularly practicing personal oral hygiene measures – for instance, daily toothbrushing with fluoridated toothpaste – to remove pathogenic biofilm from the tooth surfaces, as well as by diets rich in fermentable carbohydrates. Impairing or disturbing the attachment of bacteria can be an effective way to reduce the risk of dental caries [25].

Thus, in the present study, the growth of *S. mutans* clinical isolates was investigated in sugar-rich and sugar-free environments. The results demonstrated that with the addition of 5% sucrose, bacteria showed a greater tendency to form biofilm.

Among the gtf isoenzymes, more studies have been done on *gtfB*, because it is involved in the production of insoluble glucans and are necessary in the sucrose-dependent binding of *S. mutans* cells to hard surfaces [6], [7], [8], [9]. Therefore, in this research, the effect of the extract on the expression *gtfB* gene was examined. Nijampatnam et al. [26] showed that *gtfB* and *gtfC* make water-insoluble glucans which are important in the structural scaffold of biofilms. This indicates the positive effect of this gene in the process of attachment and formation of biofilm. In the present study, the effect of the extract on the expression of these genes, manifested as the reduction and removal of biofilm, was measured based on molecular and phenotypic tests, showing that reducing the expression of these genes reduced the biofilm by plate assay in general. Our results showed that *S. mutans* has the highest sensitivity to the extract at a concentration of 3 µg ml.

In light of the results obtained here, the antimicrobial effects of the fruite wall of acorn merits greater study. In future research, the antimicrobial compounds in fruite wall of acorncan be identified through different chemical analysis methods. Also, after examining the pharmacological effects of the purified substance in laboratory tests and clinical trials, extracts of fruite wall of acorn might have potential in the treatment and prevention of dental caries and other oral diseases/infections. 

## Conclusions

The results of biofilm measurement and reduction of *gtfB* and *brpA* gene expression showed that the extract fruite wall of acorn is effective against *S.mutans*. In the next step, the *in vitro* results need to be verified in an RCT. 

## Notes

### Competing interests

The authors declare that they have no competing interests.

### Ethical statement 

The currect study was approved by ethics committee of Ilam university of medical sciences.

### Author’s ORCID

The ORCID ID of Ghafourian S is: 0000-0002-2142-1600

## Figures and Tables

**Table 1:**
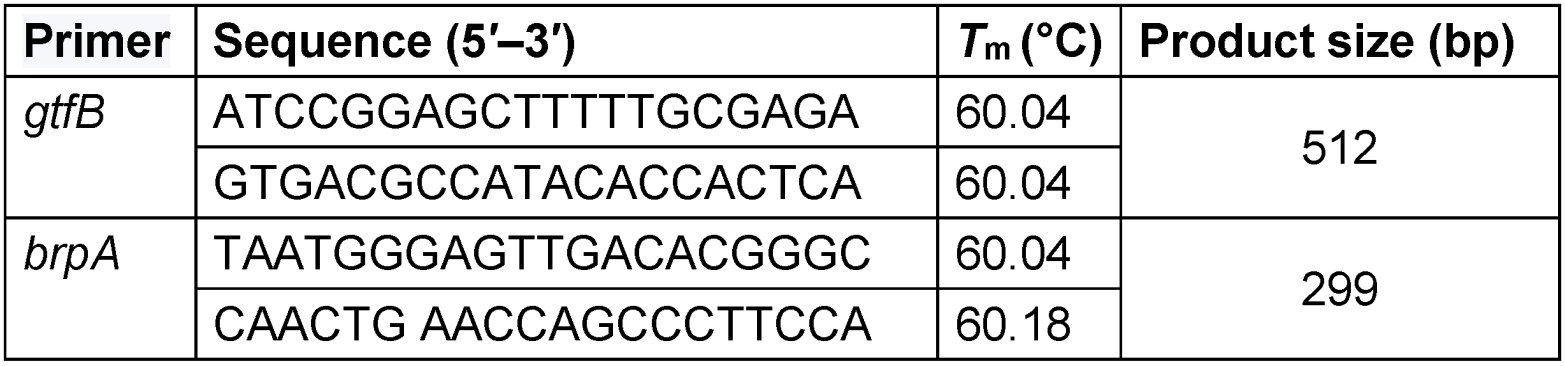
Primers used for PCR

**Table 2:**
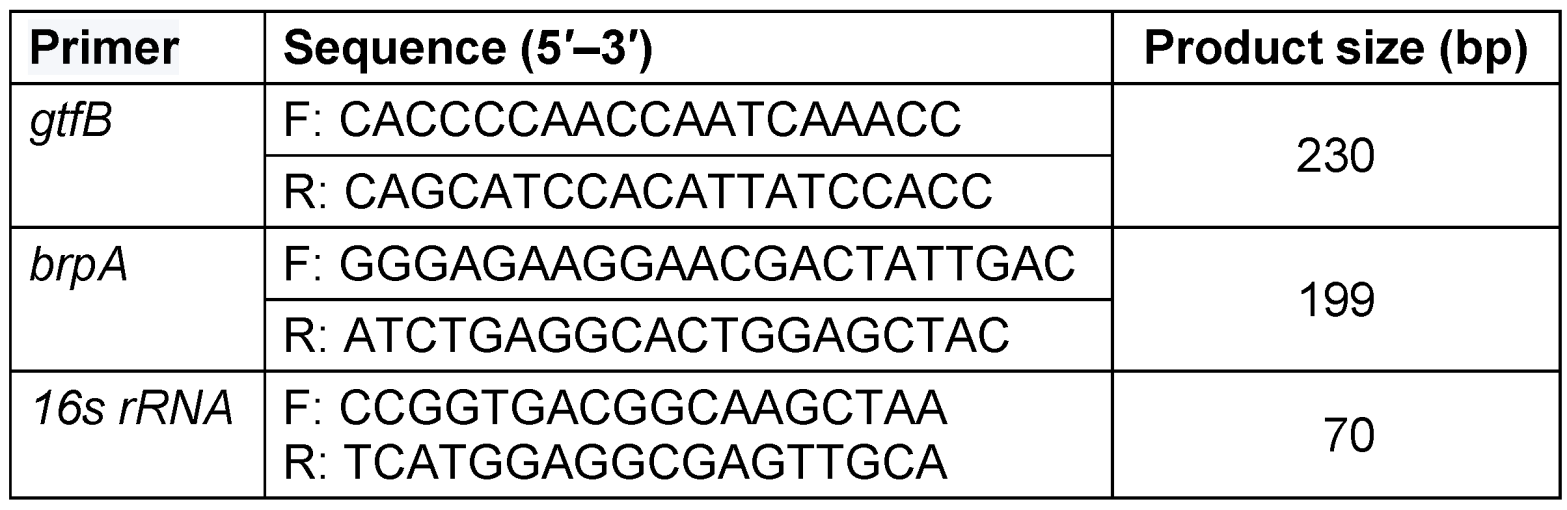
Primers used for qPCR

**Table 3:**
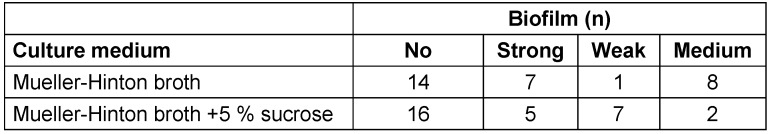
Ability of *S. mutans* to form a biofilm in two modes: 5% sucrose added and sugar-free environments (each n=30)

**Table 4:**
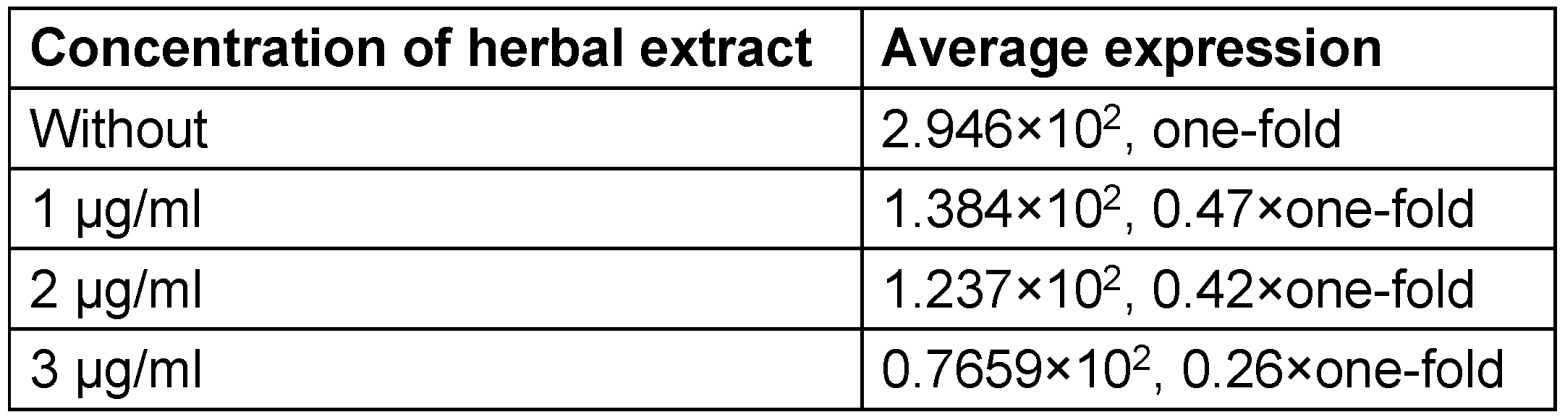
Average expression of *gtfB*

**Table 5:**
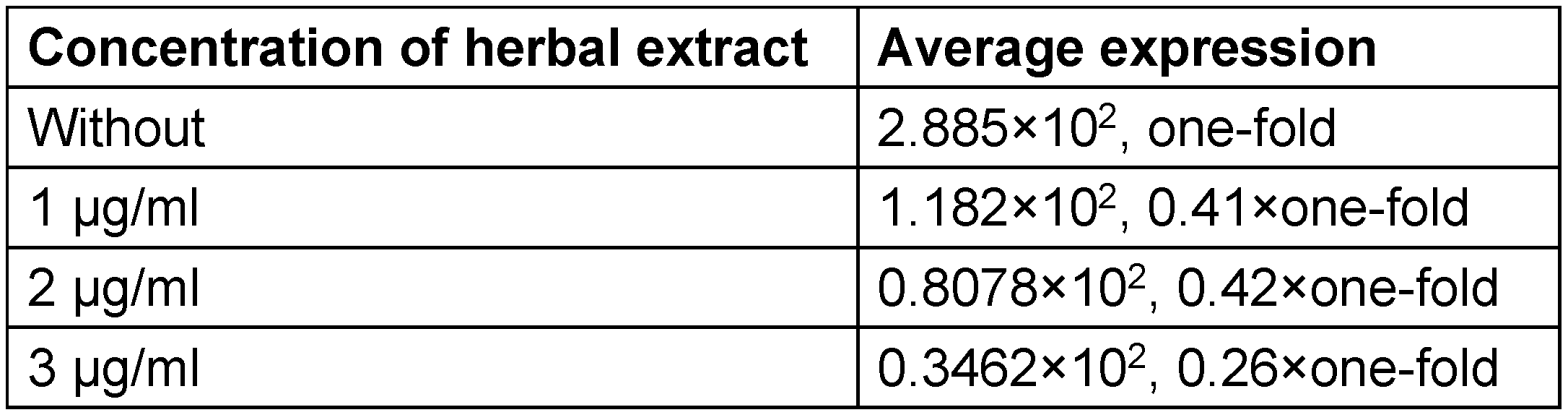
Average expression of *brpA*
